# Declining transition/transversion ratios through time reveal limitations to the accuracy of nucleotide substitution models

**DOI:** 10.1186/s12862-015-0312-6

**Published:** 2015-03-11

**Authors:** Sebastián Duchêne, Simon YW Ho, Edward C Holmes

**Affiliations:** School of Biological Sciences, The University of Sydney, Sydney, NSW 2006 Australia; Marie Bashir Institute for Infectious Diseases and Biosecurity, Charles Perkins Centre, Sydney Medical School, The University of Sydney, Sydney, NSW 2006 Australia

**Keywords:** Transition/transversion ratio, Substitution model, Virus, Substitution rate, Saturation

## Abstract

**Background:**

Genetic analyses of DNA sequences make use of an increasingly complex set of nucleotide substitution models to estimate the divergence between gene sequences. However, there is currently no way to assess the validity of nucleotide substitution models over short time-scales and with limited mutational accumulation.

**Results:**

We show that quantifying the decline in the ratio of transitions to transversions (*ti*/*tv*) over time provides an in-built measure of mutational saturation and hence of substitution model accuracy. We tested this through detailed phylogenetic analyses of 10 representative virus data sets comprising recently sampled and closely related sequences. In the majority of cases our estimates of *ti*/*tv* decrease with time, even under sophisticated time-reversible models of nucleotide substitution. This indicates that high levels of saturation are attained extremely rapidly in viruses, sometimes within decades. In contrast, we did not find any temporal patterns in selection pressures or CG-content over these short time-frames. To validate the temporal trend of *ti*/*tv* across a broader taxonomic range*,* we analyzed a set of 76 different viruses. Again, the estimate of *ti*/*tv* scaled negatively with evolutionary time, a trend that was more pronounced for rapidly-evolving RNA viruses than slowly-evolving DNA viruses.

**Conclusions:**

Our study shows that commonly used substitution models can underestimate the number of substitutions among closely related sequences, such that the time-scale of viral evolution and emergence may be systematically underestimated. In turn, estimates of *ti*/*tv* provide an effective internal control of substitution model performance in viruses because of their high sensitivity to mutational saturation.

**Electronic supplementary material:**

The online version of this article (doi:10.1186/s12862-015-0312-6) contains supplementary material, which is available to authorized users.

## Background

Accurately estimating the number of nucleotide substitutions separating gene sequences is a fundamental task in evolutionary genetics and critical to the estimation of phylogenetic relationships and divergence times. An increasingly sophisticated set of substitution models have been developed since their first appearance in the late 1960s [1], and it is generally assumed that genetic distances can be accurately estimated if the number of nucleotide substitutions per site is low [2,3]. However, other than simulation studies, there are few means of testing the validity of this assumption using empirical gene sequence data.

One simple metric of the nucleotide substitution process, and which has long been a component of substitution models [4], is the ratio of transition (purine-to-purine or pyrimidine-to-pyrimidine) to transversion (purine-to-pyrimidine or vice versa) mutations within a sequence, denoted here as *ti*/*tv*. If all of the possible pairwise nucleotide substitutions occur at the same rate, then *ti*/*tv* is expected to be 0.5 because there are twice as many possible transversions as transitions. However, it is often observed that transitions are more common than transversions. For example, the *ti*/*tv* value across the *Drosophila* nuclear genome is around 2 [5], while the equivalent value for humans is approximately 4 [6]. More extreme values are found in primate mitochondrial DNA, with reported *ti*/*tv* estimates of up to 15 in the control region [7,8] and up to ~19 in cyt *b* [9]. Importantly, the bias of transitions over transversions makes this parameter highly sensitive to mutational saturation because transitions will saturate more rapidly than transversions. As a result, *ti*/*tv* decays with the divergence times of pairs of species when mutational saturation is not taken into account [9]. For this reason, it potentially provides a useful measure of substitution model accuracy.

Viruses, particularly those with RNA genomes, are an ideal data set to compare the impact of substitution models on estimates of genetic distance because of the rapidity of their evolution. In RNA viruses, appreciable evolutionary change can occur over the time-span of human observation [10,11]. In turn, accurate model inference is central to revealing the core patterns and processes of viral evolution, and to accurately estimating the time-scale of their emergence and spread through populations [12,13]. However, the impact of site saturation is also likely to be exacerbated in RNA viruses because they possess small genomes and experience very high rates of mutation [14]. Specifically, because the viral genome is composed of a small number of genes with overlapping functions (and sequences with overlapping reading frames), then those sites that are free to vary are prone to high levels of mutational saturation. This pattern might be difficult to fully capture in the substitution models that are widely used [15]. For example, it was recently estimated that the number of multiple substitutions at a single site could be up to 39 in a portion of the HIV-1 genome sampled from 161 samples collected over 46 years, and up to 9 for the *PB2* gene of H1N1 influenza A virus from 131 samples collected over 82 years [16].

The rapid accumulation of large numbers of mutations per site potentially poses major problems for phylogenetic analyses of viruses, including estimates of their divergence times. Indeed, it is possible that site saturation might become apparent over the course of a few years, such that even the most complex of the time-reversible models may underestimate the true number of changes. However, because sequences from viruses have often been sampled from a variety of known time-points, from very shallow (e.g., within individual transmission chains) to more distant (e.g., sequences sampled globally over many decades), they also represent an ideal data set with which to document changes in the substitution process through time. In this respect, the estimate of *ti*/*tv* may represent a powerful means of investigating whether substitution models correctly account for site saturation; if a specific substitution model performs well, then estimates of *ti*/*tv* for a specific virus should be constant over time. In contrast, a decaying *ti*/*tv*, scaling negatively with time, would indicate that the substitution model does not accurately account for saturation [17].

To investigate the extent of variation in *ti*/*tv* over time, we analyzed gene sequence data from multiple RNA (n = 52) and DNA (n = 24) viruses. For each data set, we estimated *ti/tv* and the age of the root-node, which spanned a variety of time-scales (5 to ~10,000 years). To explore these evolutionary patterns more precisely, we analyzed 10 additional virus data sets in detail, spanning a diversity of virus types: *African swine fever virus* (ASFV), *Barley yellow dwarf virus* (BYDV), *Capripoxivirus* (CaPV), *Cereal yellow dwarf virus* (CYDV), *Dengue virus type 4* (DENV-4), *Ebolavirus* (EBOV), *Hepatitis B virus* (HBV), *human immunodeficiency virus 1* (HIV-1), *Rabies virus* (RABV), and HIV-2 and some closely related *simian immunodeficiency virus* (SIV) lineages (HIV-2 + SIV). The estimated evolutionary time-frames for these data sets were between approximately 50 and 900 years, allowing us to estimate evolutionary parameters over a time-span that covers many examples of viral emergence. Importantly, we used the best-fitting time-reversible model of nucleotide substitution for each data set, such that it might be expected that the impact of site saturation is minimized. This approach also allowed explicit estimation of *ti*/*tv* for each data set.

For each virus in our detailed analysis we estimated phylogenetic trees and the root-node age for the complete data, then selectively removed the most divergent lineages. This resulted in subsamples of the data that had more recent root-node ages than the complete data sets, here referred to as ‘reduced-age’ data sets; comparison between the complete and reduced-age data then allowed us to determine any changes in *ti*/*tv* over time. As well as *ti*/*tv*, we considered a set of core substitution parameters: (i) a measure of selection pressure in the form of the ratio of nonsynonymous to synonymous substitutions per site (*d*_*N*_*/d*_*S*_), (ii) the shape parameter (*α*) of the Γ-distribution of among-site rate variation, and (iii) the CG-content. These parameters allow us to pinpoint, with precision, the changing contribution of site saturation over time, while discerning the possible confounding effects of natural selection and base composition.

Estimates of *ti*/*tv* can be affected by natural selection because transversions are more likely than transitions to induce nonsynonymous (i.e. amino acid-changing) changes within proteins [18,19]. For this reason, we assessed selective constraints in our data by estimating *d*_*N*_/*d*_*S*_, a parameter that is commonly used to detect the pattern and strength of selection. However, the precise threshold value to detect positive or negative (purifying) selection depends on the demographic process [20] and on the level of sequence divergence [21]. In general, estimates of *d*_*N*_*/d*_*S*_ are expected to decay with time if purifying selection has a measurable effect, which is explained by the gradual removal of nonsynonymous polymorphisms (i.e. *d*_*N*_*/d*_*S*_ is expected to be higher toward the present because not all transient deleterious mutations will have been removed by purifying selection). Hence, if the strength and direction (positive or negative) of natural selection are constant, then the estimate of *d*_*N*_*/d*_*S*_ should be constant through time.

The presence of an increasing proportion of sites with mutational saturation should be reflected in the shape of the Γ-distribution of among-site rate variation. We can infer that the substitution model accurately accounts for increasing levels of mutational saturation if the Γ-distribution is L-shaped at deep divergence times, reflecting high levels of among-site rate variation. Hence, when estimates of the amount of evolutionary change are accurate, *α* might scale negatively with time, whereas *ti*/*tv* should remain constant. In contrast, if the substitution model does not correct for increasing levels of saturation, then the extent of among-site rate variation might appear to decrease because multiple substitutions at rapidly-evolving sites are not accurately quantified. In this case the Γ-distribution will tend to be bell-shaped, with a large value of *α*, and there will be a corresponding decline in the estimate of *ti*/*tv* over time.

Base composition is associated with estimates of *ti*/*tv*. Regions with unusually high CG-content mutate more rapidly than regions with high AT-content because of a tendency for methylation at cytosine nucleotides [22-24], so they are more prone to saturation [25,26]. Accordingly, failure to take this into account can lead to underestimation of *ti*/*tv* [27]. We investigated this potential effect by calculating CG-content over time, allowing us to assess whether any temporal trends in *ti*/*tv* were accompanied by changes in base composition.

## Methods

### Data collection

We collected nucleotide sequence data from a variety of viruses available on GenBank. For our broad-scale analyses across groups of viruses, we collected 76 data sets of nucleotide sequences from different viruses (details provided in Additional file 1: Table S1). To minimize the impact of phylogenetic non-independence, we randomly selected a single data set from each virus genus for which several data sets were available. For our detailed analysis of the substitution process, we analyzed gene sequences of 10 viruses selected to represent different categories of the widely used Baltimore classification. Within these viruses we selected individual gene sequences commonly used in phylogenetic studies: (i) the *p54* gene of *African swine fever virus* (ASFV) (double-strand DNA (dsDNA) virus)); (ii) the *CP* gene of *Barley yellow dwarf virus* (BYDV) (positive-sense, single-strand RNA (+ssRNA)); (iii) the *GPCR* gene of *Capripoxivirus* (CaPV) (dsDNA), (iv) the *CP* gene of *Cereal yellow dwarf virus* (CYDV) (+ssRNA); (v) the *E* gene of *Dengue virus type 4* (DENV-4) (+ssRNA); (vi) the *GP* gene of *Ebolavirus* (EBOV) (negative-sense, single-strand RNA (–ssRNA)); (vii) the *S* gene of *Hepatitis B virus* (HBV) (dsDNA utilizing reverse transcriptase (RT)); (viii) the *env* gene of HIV-1 (ssRNA-RT); (ix) the *G* gene of *Rabies virus* (RABV) (–ssRNA); and (x) the *env* gene of HIV-2 and some closely related SIV lineages (HIV-2 + SIV) (ssRNA-RT). The number of sequences in these data sets ranged from 27 to 50, with sequence lengths ranging from 446 nucleotides (nt) in HIV-2 + SIV to 2728 nt in HIV-1 (Additional file 2: Table S2). In all cases, sequences were aligned using the Muscle algorithm [28], which were then confirmed by visual inspection.

### Estimates of root-node ages and evolutionary rates

For each data set, we estimated the age of the root-node using a Bayesian phylogenetic method implemented in BEAST v.1.8.0 [29]. We selected the nucleotide substitution model according to the Bayesian Information Criterion (BIC) in ModelGenerator v.0.851 [30]. We used the BIC because it has been shown to have higher performance than other model selection methods, such as likelihood ratio tests and other information-based criteria [31]. The best-fitting models chosen allowed the explicit estimation of *ti*/*tv*: HKY (for ASFV, BYDV, CaPV, EBOV, and HBV), HKY + Γ (for CYDV, RABV, and HIV-2 + SIV), TrN + Γ (for DENV-4), and TVM + I + Γ (for HIV-1) (Additional file 2: Table S2). In our broad-scale analyses of 76 viruses, we similarly used the substitution model with the highest BIC score. In each case we utilized the Bayesian skyline demographic model [32] and used the sampling times (years) of the sequences for calibration (i.e. tip-dating). We obtained samples from the posterior distribution every 10^3^ steps from a total of 10^8^ Markov chain steps, with the first 10% of steps discarded as burn-in. We assessed sufficient sampling by ensuring that the effective sample size for all parameters was at least 200, using the R package CODA [33].

For the BEAST analyses, we also conducted a date-randomization test to determine whether the estimates of rates and time-scales were reliable [34]. This test involves randomizing the sampling times of the sequences and re-analyzing the data several times. The estimates are considered reliable if the 95% credible intervals of the rate estimated with the randomized data sets does not include the mean rate estimated using the correct sampling times [35,36]. We conducted 10 randomizations for each data set. According to this criterion, the estimates from the 10 data sets that we used for our detailed analyses were reliable, as were 44 of the 76 data sets from our broad-scale analyses (Additional file 3: Table S3).

### Estimates of other substitution model parameters

We estimated a set of core parameters at different points in time for each virus data set in our detailed analyses. Specifically, we estimated *ti*/*tv*, the ratio of the number of synonymous to nonsynonymous substitutions per site (*d*_*N*_/*d*_*S*_), the *α* shape parameter of the Γ-distribution of among-site rate variation, and CG-content in the 10 virus data sets that comprised our case study. To achieve this, we removed the lineages that led to the root-node of the maximum clade credibility (MCC) tree estimated in BEAST from the complete data set (Additional file 4: Figure S1). This resulted in subsampled data sets with younger root-node ages than the complete data, which we refer to as ‘reduced-age’ data sets. The estimates from the complete and reduced-age data sets are not independent, precluding the application of standard statistical tests. However, this analysis does allow us to illustrate evolutionary patterns through time within closely related viruses.

We estimated *ti*/*tv*, *d*_*N*_/*d*_*S*_, *α*, and the CG-content for the complete and the reduced-age data sets, using the CODEML program from the PAML v. 4.7 package [37]. The tree topologies were fixed according to the maximum likelihood trees estimated in GARLI [38], using the substitution models previously selected in ModelGenerator. We set five discrete rate categories for the Γ-distribution and used a codon model that assumed a single value of *d*_*N*_/*d*_*S*_ for all branches in the tree. These settings are convenient because the values of *ti*/*tv*, *d*_*N*_/*d*_*S*_, *α*, and CG-content can be optimized simultaneously.

A potential shortcoming of our approach is that the reduced-age data sets always have fewer sequences than the complete data, which can confound the inference of temporal patterns in the parameter estimates. To address this, we randomly removed a set of sequences from each data set, while maintaining the original root-node age. This procedure led to data sets of equivalent size to the reduced-age data sets described above. For each virus we generated 100 randomly subsampled data sets and conducted the same analysis in CODEML. The estimates from the randomly subsampled data sets are the expected values obtained as a result of a reduction in the number of sequences, while maintaining the root-node age, thereby allowing us to assess the effect of removing only the most divergent sequences. This method is comparable to generating a ‘null’ range of values for each parameter estimate. Hence, we considered that temporal trends were present in the data only if the estimates from the reduced-age data sets were not within the range of the estimates obtained with the randomly subsampled sequences.

### Simulations of different levels of saturation and the shape of the Γ-distribution

We conducted a set of simulations to further investigate the behavior of the *α* parameter of the Γ-distribution under different levels of mutational saturation. Specifically, we simulated the evolution of DNA sequences along 100 phylogenetic trees of 10 taxa using Phangorn v. 1.99 [39]. We obtained four data sets per tree, each representing a different level of sequence divergence. To do this we scaled the total tree length to 1 substitution per site (subs/site) and then we multiplied the branch lengths by 5, 10, and 15. The sequence data simulated along long trees in this manner are expected to display a large amount of mutational saturation. The simulations produced sequence alignments of 500 nucleotides. We set the value of *ti*/*tv* to 4 and we included among-site rate variation by specifying a Γ-distribution with an *α* parameter of 0.4. We analyzed our simulated data by optimizing the branch lengths, the substitution rate matrix, and *α* using maximum likelihood in Phangorn.

### Broad-scale analyses of *ti*/*tv* in 76 viruses

For our broad-scale analysis, we considered the mean estimate of *ti*/*tv* and the root-node age estimated in BEAST for each virus. We analyzed DNA and RNA viruses separately to account for differences in their evolutionary dynamics (i.e., because of their reliance on an error-prone polymerase, RNA viruses are generally expected to exhibit higher substitution rates than DNA viruses). For our statistical analysis we excluded 32 data sets (11 DNA and 21 RNA virus) that did not pass the date-randomization test because the estimates of the root-node age might be unreliable (Additional file 3: Table S3). We fitted robust linear models for *ti*/*tv* as a function of the root-node age on a log_10_ scale. This method is more appropriate than ordinary linear regressions for our data because of the small number of points.

## Results

Estimates of mean evolutionary rates in the 10 viruses in our case study ranged across two orders of magnitude, from 2.39 × 10^−5^ nucleotide substitutions per site, per year (subs/site/year) for the complete BYDV (+ssRNA) data set to 2.35 × 10^−3^ subs/site/year for the reduced-age CYDV (+ssRNA) data set (Table 1). Although there was some overlap in the 95% credible intervals (CI) between the rates estimated from the reduced-age and the complete data sets, the reduced-age data sets had consistently higher mean rate estimates. One likely explanation for the temporal differences in the rate estimates is that the number of multiple substitutions is underestimated for deep sections of the tree, so that the rate appears lower.

**Table 1:** **Evolutionary rate estimates for the complete and the reduced-age data sets of 10 viruses**

	**Mean rate and 95%** **credible interval (substitutions/site/year)**	
**Virus data set and number of sequences (reduced-age/complete data)**	**Complete data set**	**Reduced-age data set**
ASFV (22/50)	3.61 × 10^−5^ (3.31 × 10^−5^ – 4.29 × 10^−5^)	4.58 × 10^−5^ (3.39 × 10^−5^ – 1.40 × 10^−4^)
BYDV (17/27)	2.39 × 10^−5^ (1.94 × 10^−5^ – 3.46 × 10^−3^)	2.81 × 10^−5^ (2.12 × 10^−5^ – 4.62 × 10^−5^)
CaPV (20/37)	6.50 × 10^−5^ (6.04 × 10^−5^ – 7.11 × 10^−5^)	8.56 × 10^−5^ (6.17 × 10^−5^ – 2.21 × 10^−4^)
CYDV (45/76)	1.79 × 10^−3^ (1.75 × 10^−4^ – 7.89 × 10^−3^)	2.35 × 10^−3^ (2.57 × 10^−4^ – 761 × 10^−3^)
DENV-4 (32/49)	1.12 × 10^−3^ (5.38 × 10^−5^ – 4.09 × 10^−3^)	1.51 × 10^−3^ (7.48 × 10^−5^ – 4.23 × 10^−3^)
EBOV (17/32)	3.89 × 10^−4^ (1.92 × 10^−4^ – 7.15 × 10^−4^)	4.75 × 10^−4^ (1.99 × 10^−4^ – 1.11 × 10^−3^)
HBV (15/34)	2.06 × 10^−4^ (1.51 × 10^−4^ – 3.90 × 10^−4^)	2.34 × 10^−4^ (1.54 × 10^−4^ – 6.78 × 10^−4^)
HIV-1 (17/29)	3.21 × 10^−3^ (2.59 × 10^−3^ – 4.15 × 10^−3^)	4.06 × 10^−3^ (2.62 × 10^−3^ – 8.59 × 10^−3^)
RABV (16/48)	4.56 × 10^−4^ (3.48 × 10^−4^ – 6.64 × 10^−4^)	5.33 × 10^−4^ (3.36 × 10^−4^ – 1.05 × 10^−3^)
HIV-2 + SIV (18/30)	1.03 × 10^−3^ (6.79 × 10^−4^ – 1.56 × 10^−3^)	1.42 × 10^−3^ (7.00 × 10^−4^ – 4.54 × 10^−3^)

**Figure 1:**
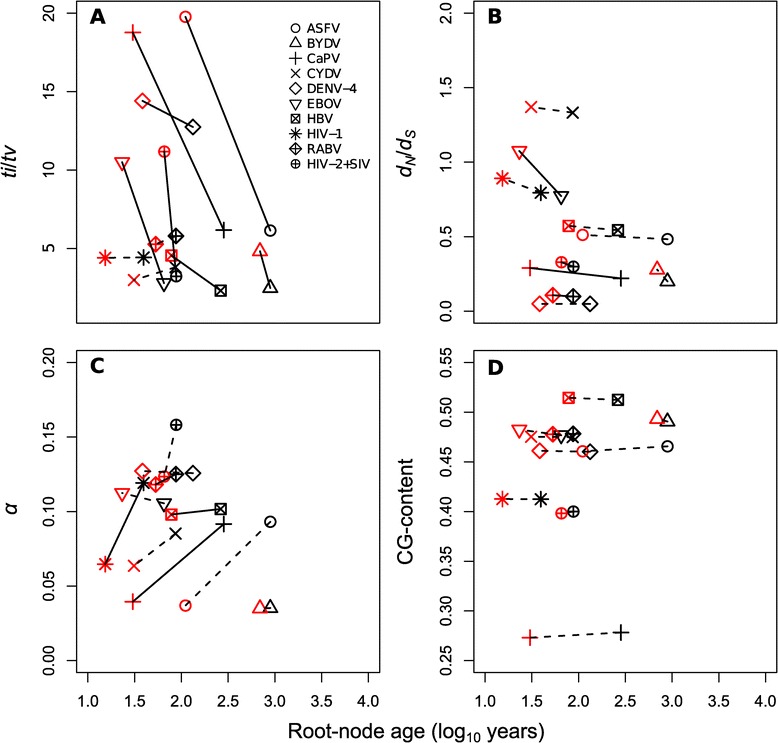
**Estimates of key parameters plotted against root-node age for 10 representative viruses. (A)** *ti*/*tv*, **(B)** *d*_*N*_ */d*_*S*_, **(C)** shape parameter *α* of the Γ-distribution, and **(D)** CG-content. The symbols represent the different viruses: *African swine fever virus* (ASFV), *Barley yellow dwarf virus* (BYDV), *GPCR* gene of *Capripoxivirus* (CaPV), *CP* gene of *Cereal yellow dwarf virus* (CYDV), *Dengue virus type 4* (DENV-4), *Ebolavirus* (EBOV), *Hepatitis B virus* (HBV), HIV-1, *Rabies virus* (RABV), and HIV-2 and some closely related SIV lineages (HIV-2 + SIV). Black symbols correspond to the complete data sets, while red symbols correspond to the reduced-age data sets in which we removed the most divergent lineages. Lines show the differences in the estimates between the complete and the reduced-age data sets, and do not represent regressions. Dashed lines correspond to estimates that are not considered to differ between the complete and reduced-age data sets, which is assessed by estimating the parameters with random subsamples of the data (see Additional file 2: Table S2).

The abbreviations correspond to those in Figure 1.

Estimates of *ti*/*tv* declined over time in most of our virus data sets (Figure 1A). The reduced-age data sets of ASFV, CaPV, EBOV and HIV-2 + SIV had estimates of *ti*/*tv* of 19.76, 18.77, 10.50, and 11.17, respectively; these were more than three-fold higher than those for the complete data sets, at 6.14, 6.17, 2.79, and 3.22, respectively. This pattern was especially pronounced in HIV-2 + SIV and EBOV. In the case of HIV-2 + SIV the nucleotide substitution rate was 1.42 × 10^−3^ subs/site/year (95% CI: 7.00 × 10^−4^ – 4.54 × 10^−3^) for the reduced-age data set and 1.03 × 10^−3^ subs/site/year (95% CI: 6.79 × 10^−4^ – 1.56 × 10^−3^) for the complete data set (Table 1), with corresponding mean root-node ages of 65 years (95% CI: 43 – 73) and 88 years (95% CI: 77 – 108). Similarly, the nucleotide substitution rate for EBOV was 4.75 × 10^−4^ subs/site/year (95% CI: 1.99 × 10^−4^ – 1.11 × 10^−3^) for the reduced-age data, and 3.89 × 10^−4^ subs/site/year (95% CI: 1.92 × 10^−4^ – 7.15 × 10^−4^) for the complete data, with corresponding mean root-node ages of 23 years (95% CI: 21 – 30) and 65 years (95% CI: 33 – 70). This indicates that there is a measurable amount of saturation within decades for these viruses.

In ASFV and CaPV the disparities between the estimates of the mean root-node ages for the reduced-age and the complete data sets were greater, at approximately 783 and 253 years, respectively, suggesting that high levels of saturation are attained more slowly in these viruses than in HIV-2 + SIV and EBOV. These estimates also corresponded to lower substitution rate estimates for these viruses, of 3.61 × 10^−5^ subs/site/year (95% CI: 3.31 × 10^−5^ – 4.29 × 10^−5^), and 6.50 × 10^−5^ subs/site/year (95% CI: 6.04 × 10^−5^ – 7.11 × 10^−5^) for the complete data sets of ASFV and CaPV, respectively. In the reduced-age data set of DENV-4, the estimate of *ti*/*tv* was 14.40, which is 1.13 times that for the complete data set, at 12.74, suggesting only a modest decline over time. In contrast, the HIV-1, CYDV, and RABV data sets did not display a decline in the estimate of *ti*/*tv* over time, with estimates for the reduced-age data that were in the range of those obtained with random subsamples (Additional file 2: Table S2 and Additional file 5: Table S4).

Importantly, we did not find evidence of strong temporal patterns of selection pressure in the viruses analyzed here. Only in the case of EBOV and CaPV was there a modest decline in the estimate of *d*_*N*_/*d*_*S*_ over time, for which the differences in the estimates for the complete and the reduced-age data sets were 0.3 and 0.07, respectively. For the remaining data sets we did not find a measurable trend in *d*_*N*_/*d*_*S*_, with estimates that were in the range of values obtained with random subsamples of the data (Figure 1B).

**Figure 2:**
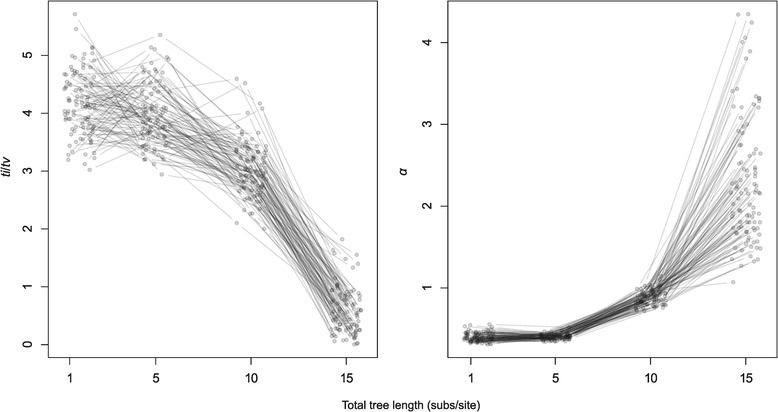
**Estimates of** ***ti*** **/** ***tv*** **and of the shape parameter,** ***α*** **, of the Γ-distribution of among-site rate variation plotted against total tree length (subs/site) for simulated data.** Each line corresponds to a phylogenetic tree and the points are the maximum likelihood estimates for data simulated on trees with different lengths (x-axis). For clarity, the points have been jittered along the x-axis.

Our simulations of the behavior of *α* (a measure of the extent of among-site rate variation) illustrate the expectation of this parameter under different levels of saturation and its relationship with *ti*/*tv*. We found that increasing the tree length led to a decrease in the estimate of *ti*/*tv* and an increase in that of *α* (Figure 2). We use tree length as a proxy for divergence time, such that long trees represent deep evolutionary time-scales. The trend in the estimates of these parameters is not necessarily linear. In particular, the estimate of *ti*/*tv* appeared to decrease slowly over time for our simulations based on tree lengths of 10 or less. In a few simulations, the estimate of this parameter increased slightly before sharply decreasing. Similarly, the estimate of *α* appeared to increase slowly for data sets simulated on trees of length 5 or less. We also observed a large variation in the estimate of *α* for deep evolutionary time-scales. Although the relationship between these parameters is complex, these simulations validate our general prediction that failure to account for mutational saturation can lead to an increase in the estimate of *α* over time and a decline in that of *ti*/*tv*. These results also show that these parameters can be estimated accurately only for very recent evolutionary time-scales.

The estimate of *α* increased over time for CaPV, HIV-1, HBV, and RABV. In CaPV and HBV we also found that the estimates of *ti*/*tv* declined over time, suggesting that the substitution models did not accurately account for mutational saturation in the complete data sets (Figure 1C). For example, the estimate of *α* for the complete CaPV data set was nearly two-fold higher than that for the reduced-age data set. This result is consistent with increasing numbers of saturated sites over time that are not correctly quantified by the substitution model. We did not detect this trend in other viruses (Additional file 2: Table S2 and Additional file 5: Table S4).

Base composition differed considerably among the viruses studied here. The lowest CG-content was observed in CaPV (0.27), while the HBV data set had the highest CG-content (0.51). However, this parameter did not display variation over time for any of the data sets (Figure 1D). The expectation that viruses with high CG-content should experience very rapid saturation, with large variation in the estimate of *ti*/*tv* over time, was not supported in the analysis of these particular viral data sets. For example, the estimate of *ti*/*tv* for the CaPV data set has a strong negative association with time, but this data set has a very low CG-content. A possible reason for this finding is that rates of evolution in viruses are largely governed by selective constraints, such that CG-content is a poor predictor of rate variation in viruses.

**Figure 3:**
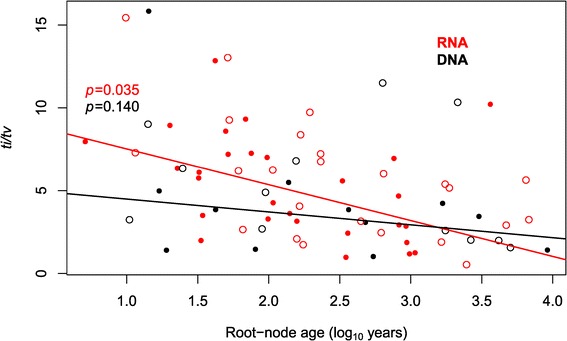
**Estimates of** ***ti*** **/** ***tv*** **plotted against root-node age in a broad-scale analyses of DNA (n = 52) (black) and RNA (red) (n = 24) viruses.** The points represent individual virus data sets and the lines represent the regressions. Solid circles correspond to data sets that passed the date-randomization test, whereas empty circles are those that failed the test.

Importantly, our broad-scale analysis of substitution patterns across a diverse array of viruses also revealed a negative association between the estimate of *ti*/*tv* and the age of the root-node in the case of RNA viruses, again indicative of site saturation. Estimates for 32 data sets were found to be unreliable according to the date-randomization test [33], such that they were excluded from the linear regression. For the remaining viruses, the slope coefficients for *ti*/*tv* as a function of the root-node age on a log_10_ scale were -0.78 and -2.16 for DNA and RNA viruses, respectively (Figure 3). Notably, the slope term was significant for RNA viruses (*p* = 0.035), but not for DNA viruses (*p* = 0.14), suggesting that site saturation occurs on short time-scales for the more rapidly evolving RNA viruses.

## Discussion

Our broad-scale analyses reveal a general decline in estimates of *ti*/*tv* over time. The most important aspect of this trend is that it is present in viruses sampled over very short time-frames, in which few substitutions have accumulated and genetic distances are estimated using models of nucleotide substitution that were found to be the best-fitting to the data. Importantly, although this pattern could in theory be attributed to the effect of purifying selection and non-stationary base composition, the temporal variation in both of these factors is insufficient to explain the decline in *ti*/*tv* over short time-frames. Consequently, a more probable explanation for the decline in the estimate of *ti/tv* over short time-frames is that this parameter is systematically underestimated, even under the most complex time-reversible substitution models, because viruses attain high levels of saturation very rapidly. In this respect, saturation should occur sooner in rapidly evolving RNA viruses than in more slowly evolving DNA viruses. This contention is supported by our broad-scale analysis, for which the decline in *ti/tv* over time was more pronounced (and statistically significant) for the rapidly evolving RNA viruses than for the more slowly evolving DNA viruses. In this context it is important to note that there might be some gene-specific exceptions to this trend. For example, a highly variable gene in a DNA virus might undergo mutational saturation rapidly and display a stronger temporal decline in *ti*/*tv* than a highly conserved gene in an RNA virus. In this context it is important to note that our aim was to explore general trends using viral genes commonly used in phylogenetic analysis, rather than to perform an exhaustive analysis of all virus-gene combinations.

In some cases the *α* parameter of the Γ-distribution was nearly two-fold higher for the complete data sets than for the reduced-age data, which probably occurs because many sites are under strong selective constraints and only a small proportion of sites are free to vary. The substitution models fail to account for increasing levels of saturation at these sites over time, such that the degree of among-site rate variation is underestimated. In CaPV and HBV, for example, the estimates of *α* increased while that of *ti*/*tv* declined over time. This suggests that the inclusion of a Γ-distribution of among-site variation is sometimes insufficient to allow accurate estimation of the number of multiple substitutions. Therefore, widely used substitution models might be inadequate for many virus data sets, even for those sampled in the very short term (over decades). More generally, the results presented here highlight the complex relationship between key substitution parameters, and indicate that more work is needed to reveal their determinants.

The importance of substitution model inadequacy in evolutionary studies has long been recognized [40-44]. Failure to correct for mutational saturation is problematic because it can lead to incorrect estimation of evolutionary rates and time-scales [12,15,45], and particularly a tendency to underestimate the lengths of the deep branches in the tree. In molecular clock analyses, this can cause an underestimation of the root-node age, a phenomenon known as ‘tree compression’ [46]. Relaxed molecular-clock models attempt to compensate for this effect by assigning lower rates of evolution to deep sections of the phylogeny rather than to the more recently diverging lineages, as shown in our results. However, empirical studies show the root-node age can still be underestimated with these models [46]. For this reason, the evolutionary time-scales for many viruses might be underestimated, even for those with recent origins, which is critical to understand their emergence. One potential solution is to use experimentally determined evolutionary models, in which rates of each mutation type and site-specific selection pressures for the gene of interest are measured using appropriate laboratory assays including deep sequencing [47]. This promising method has been shown to produce considerable improvements in phylogenetic analyses of an influenza nucleoprotein gene. However, its implementation for other viruses will require intensive experimental effort. Alternatively, it may be possible to identify genomic regions within which site saturation occurs more slowly, such as those with more slowly decaying *ti*/*tv*, and preferentially use these to estimate divergence times. A detailed characterisation of the dynamics of *ti/tv* across genomic regions is beyond the scope of this paper, but the methods described here can be used for this purpose in future studies.

## Conclusions

Our results show that estimates of *ti*/*tv* are highly sensitive to mutational saturation. As a consequence, they can be used as an indicator of substitution model performance in rapidly evolving viruses. If the estimate of this parameter decays over time, the substitution model is likely to be underestimating the true number of multiple substitutions, such that it has poor performance. Our method of estimating *ti*/*tv* for subsamples of the data is convenient for this purpose, and provides a simple internal test of the substitution process. It might also be useful to characterize the short-term estimates of *ti/tv* for different viruses, which could be considered as the expectation for particular virus groups. Empirical data sets that produce unreliable estimates of the amount of genetic change, and consequently, rates of evolution and evolutionary time-scales, can be detected on the basis of whether the observed estimate of *ti*/*tv* matches the expected.

## Availability of supporting data

The data sets and computer code supporting the results of this study are available in the GitHub repository, doi:10.5281/zenodo.14824

## Additional files

Additional file 1: Table S1. GenBank accession numbers of all nucleotide sequences collected for this study. Additional file 2: Table S2. Substitution models used in BEAST and the parameter estimates from CODEML for the complete and the reduced-age data sets for the 10 viruses used in the detailed analyses. The asterisk (*) indicates the estimates for the reduced-age data that differed from those obtained with random subsamples. Additional file 3: Table S3. Estimates of *ti*/*tv* and root-node age, and result of the date randomization test for the broad-scale analyses. Additional file 4: Figure S1. Maximum clade credibility trees for the 10 virus data sets used in the detailed analyses. The reduced-age data sets were obtained by removing the sequences labeled in red. Additional file 5: Table S4. Temporal trends for different parameters from the analyses of the 10 virus data sets. Estimates that were higher for the reduced-age than for the complete data sets are considered to decrease over time, while the opposite case is considered as an increase over time. Parameters with no change over time are those in which the estimates with the reduced-age data were within the range of values obtained with 100 random subsamples.

## Abbreviations

ASFV: *African swine fever virus*
BIC: Bayesian Information Criterion
BYDV: *Barley yellow dwarf virus*
CaPV: *Capripoxivirus*
CYDV: *Cereal yellow dwarf virus*
d_N_: Number of nonsynonymous substitutions per nonsynonymous site
d_s_: Number of synonymous substitutions per synonymous site
DENV-4: *Dengue virus type 4*
EBOV: *Ebolavirus*
HBV: *Hepatitis B virus*
HIV: *Human immunodeficiency virus*
MCC: Maximum clade credibility
RABV: *Rabies virus*
SIV: *Simian immunodeficiency virus*
ti/tv: Transition/transversion ratio
