# Supplementary material for: Declining transition/transversion ratios through time reveal limitations to the accuracy of nucleotide substitution models
Source: BMC Evol Biol. 2015 Mar 11;15:36. doi: 10.1186/s12862-015-0312-6 (PMC4358783; doi:10.1186/s12862-015-0312-6)
Supplement: Additional file 4: Figure S1. — Maximum clade credibility trees for the 10 virus data sets used in the detailed analyses. The reduced-age data sets were obtained by removing the sequences labeled in red. [file 12862_2015_312_MOESM4_ESM.pdf]

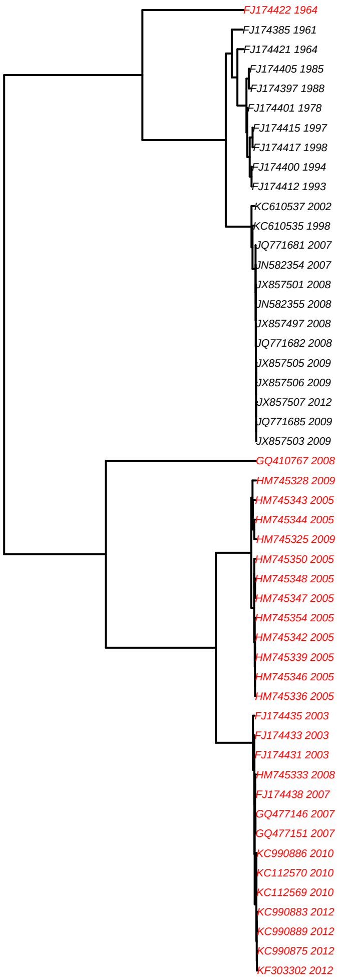

ASFV subsampled

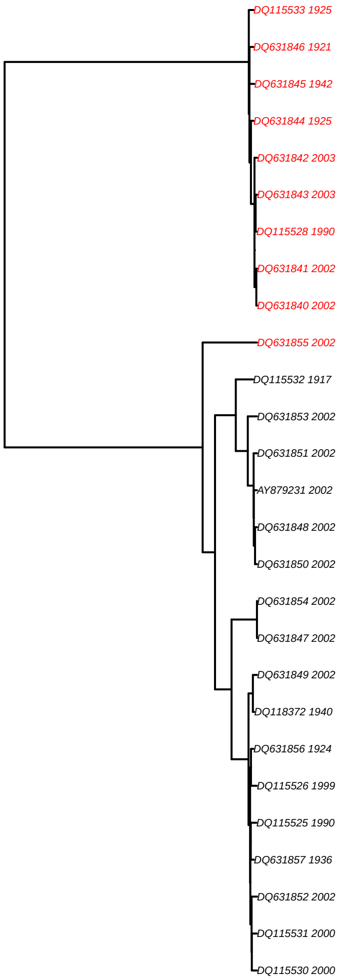

BYDV subsampled

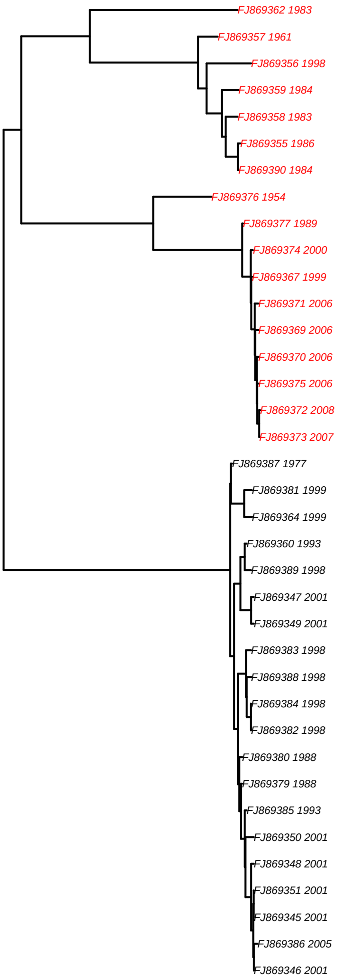

CaPV subsampled

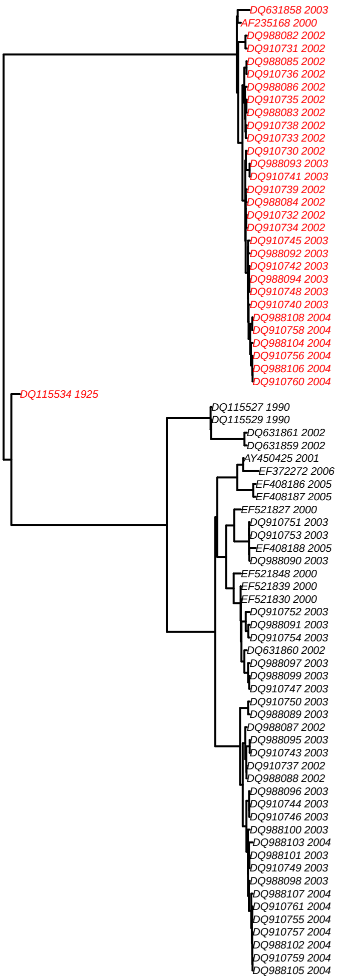

CYDV subsampled

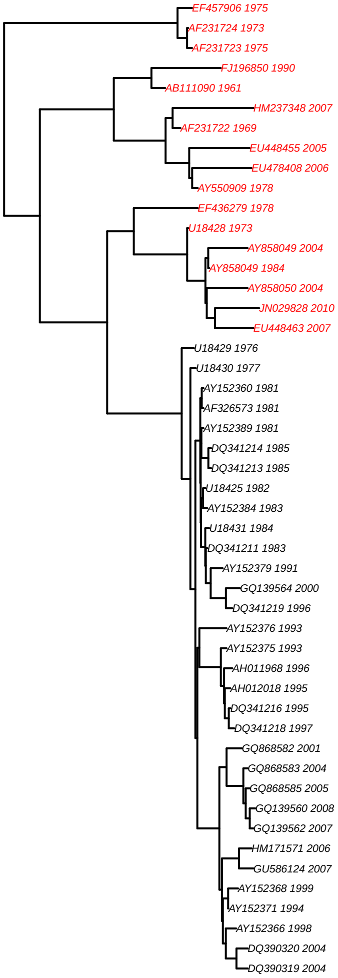

DENV−4 subsampled

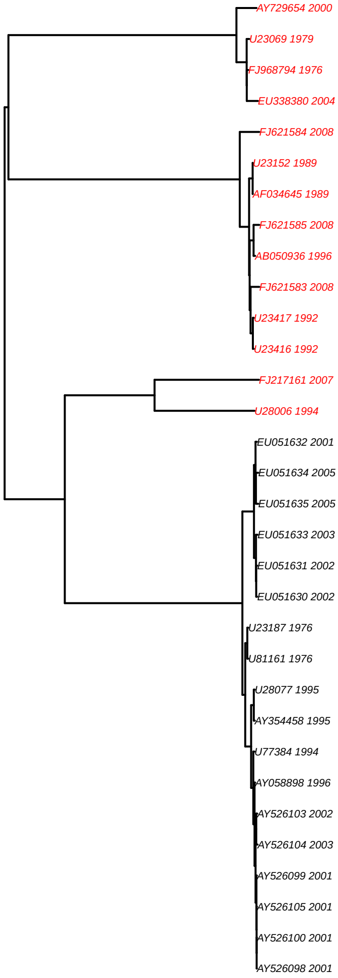

EBOV subsampled

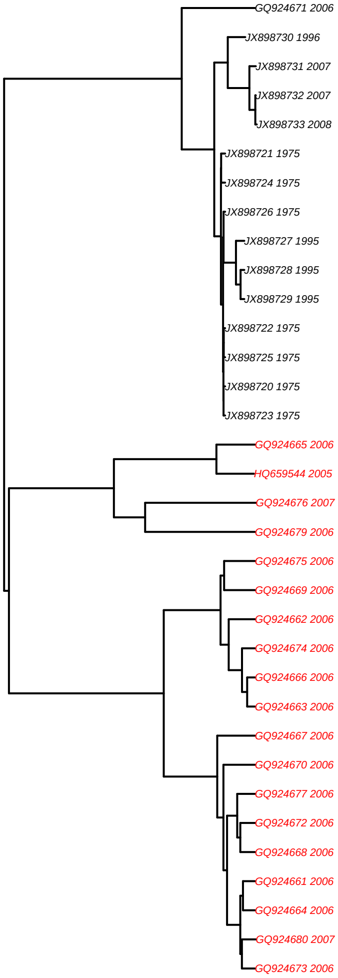

HBV subsampled

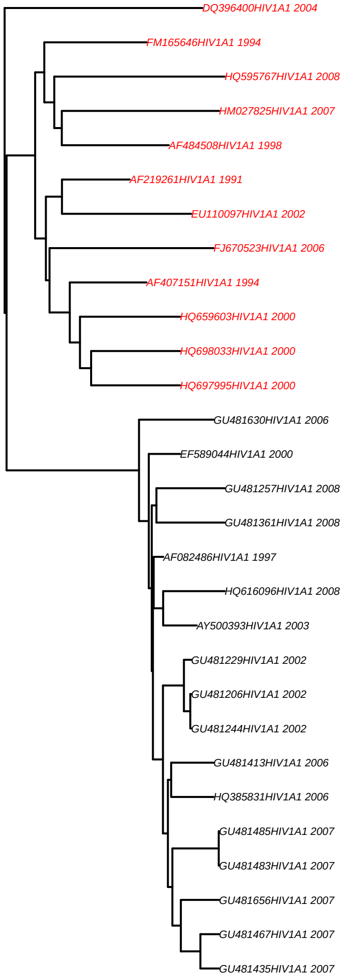

HIV−1 subsampled

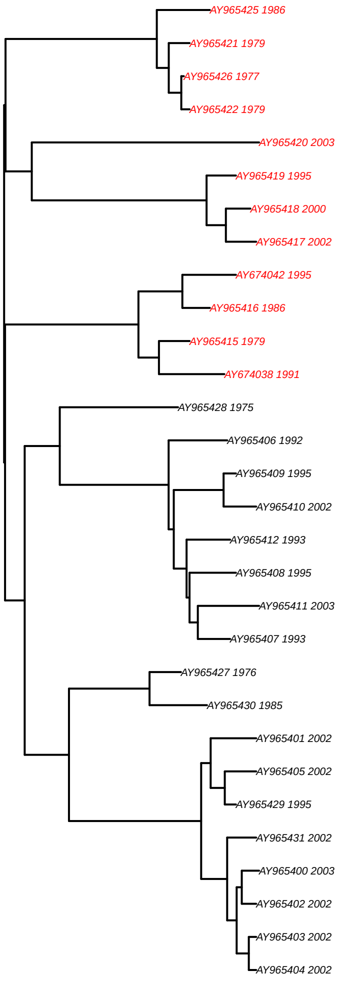

HIV−2+SIV subsampled

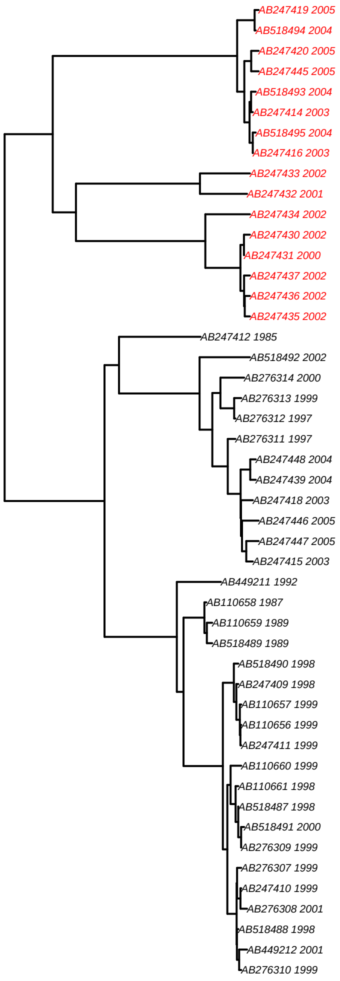

RABV subsampled
